# Z-Mammaplasty: A Novel Concept in Mastopexy

**Published:** 2011-06-27

**Authors:** Devinder P. Singh, Antonio J. V. Forte, John G. Apostolides, Hamid R. Zahiri, Jeffrey Stromberg, Nivaldo Alonso

**Affiliations:** ^a^Division of Plastic Surgery, University of Maryland School of Medicine, Baltimore, MD; ^b^Division of Plastic Surgery, Yale University School of Medicine, New Haven, CT; ^c^Division of Plastic Surgery, Johns Hopkins School of Medicine, Baltimore, MD; ^d^Division of General Surgery, University of Maryland School of Medicine, Baltimore, MD; ^e^Division of Plastic Surgery, University of Sao Paulo School of Medicine, Sao Paulo, Brazil

## Abstract

**Background:** The inverted-T technique is the most popular skin pattern used for mastopexy, but short scar variations have increased in popularity over recent years. With respect to nipple elevation, superior, superomedial, medial, inferior, lateral, and central pedicle designs have been described. **Objectives:** We introduce a novel concept for mastopexy, the glandular Z-mammaplasty, and assess its anatomic and technical feasibility. **Methods:** Glandular Z-plasty was performed on 15 human female cadavers. Various parameters were measured pre- and postoperatively to assess degree of ptosis and subsequently compared by student *t* test. **Results:** Average pre and postoperative breast width (28.5 ± 4.7 cm, 26.7 ± 3.2 cm, *P* = .009), breast length (25 ± 6.6 cm and 21.8 ± 4.3 cm *P* = .005), breast height (10.7 ± 3.7 cm and 9.5 ± 2.9 cm, *P* = .02), and ptosis degree (1.9 ± 0.9 cm and 0.3 ± 0.5 cm, *P* < .0001) were determined. Inferior limb transposition moved the nipple closer to the sternal notch and sternum midline an average of 5.3 ± 2.2 cm and 2.4 ± 1.7 cm, respectively. The average Z-plasty degree was 34.5 ± 8.2°. The average central limb length was 8.7 ± 2.1, and the average pedicle width was 5.4 ± 0.8 cm. Buttress support of the nipple was accomplished by caudal transposition of the superior Z-plasty flap and its inset below the nipple. **Conclusion:** We demonstrate that glandular Z-mammaplasty is indeed feasible. The grade of ptosis was statistically significantly improved, with the nipple moving superiorly an average of 5.3 cm in our study group.

Today's plastic surgeon faces a continuous demand to correct the effects of aging and gravity in an attempt to optimize the aesthetics of the female breast. This challenge has driven the innovations for surgical techniques that produce consistent and effective results. The surgical goal is to create breast lift to counteract the long-term effects of gravity and skin and breast tissue modification to manage the tissue changes that accompany the aging process.^1^ Several proven techniques exist for mastopexy, all of which involve a variation of nipple elevation combined with a skin reduction pattern. With respect to nipple elevation and viability, many different pedicles have been described, including superior, superomedial, medial, inferior, lateral, and central pedicles. The large number and variety of pedicle location and design lend to individual preferences and variations amongst plastic and reconstructive surgeons. There lacks a consensus as to which pattern yields superior results, aesthetically, functionally, and with regard to duration of lift. Current demands to reduce the size of scar after mammaplasty have motivated skin sparing approaches. Rohrich et al^2^ report that the inverted-T technique is the most popular approach but short scar variations have increased in popularity over recent years. The successful mastopexy combines an aesthetically pleasing skin pattern to reduce breast envelope size with a breast tissue pedicle to maintain nipple viability and support breast lift. The lack of consensus regarding skin pattern and pedicle type inspired us to search for alternative approaches to the mastopexy, with the aim of achieving reliable and successful functional and aesthetic results.^3^^-^5 Our goal is to introduce a novel technique for mastopexy and to assess the anatomic and surgical feasibility of this concept: the glandular Z-mammaplasty.

## METHODS

Fifteen human cadaver dissections of the female breast were performed (2 formalin-preserved and 13 fresh) using 2.5x loupe magnification. The surgical concept for the Z-mammaplasty is to combine current successful and commonly used skin patterns with our new technique for glandular reorientation and lift. We performed the glandular Z-plasty using both vertical and periareolar skin incisions using fresh cadavers. Both skin patterns were followed by a Z-plasty in the breast gland and fat. The nipple was necessarily included on the inferior flap (by definition making it a superomedial pedicle). It is necessary to maintain the nipple on the inferior flap, to allow Z-plasty to achieve the primary goal of mastopexy, to raise nipple position. Following the dissection of breast tissue, the flaps of the z-plasty are transposed, causing the inferior segment containing the nipple to be lifted and buttressed by the transposed superior flap, which now lies at the inferior base of the breast. The following variables were measured both before and after glandular Z-plasty and compared by student *t* test: breast width, length, height, sternal-to-nipple distance, fold-to-nipple distance, midline-to-nipple distance, degree of ptosis (0—nipple above inframammary fold, 1—nipple at fold, 2—nipple below fold but above lowest pole, and 3—nipple at the lowest pole of the breast), areolar diameter, sternal notch-to-point A, length of vertical incision, distance above the fold where incision terminates, and length of circular incision for periareolar approach.

The *breast width* was defined as the distance between the anterior axillary line and the chest wall midline at the level of the nipple. The *breast length* was defined as the distance between a line along the breast meridian from the second rib to the fold through the nipple. The *breast height* was defined as the distance between the nipple and the fold.

Intraoperatively, we determined the need for fixation, the Z-plasty angle, the pedicle width, central limb length of the Z, and number of perforators visualized. Because of prominent rigor mortis in the fresh group, cadavers could not be positioned in the seated position for markings or for intraoperative assessment. Therefore, the breasts were pushed caudally to simulate gravity during the measurement of the sternal-nipple distance.

**Figure 1:**
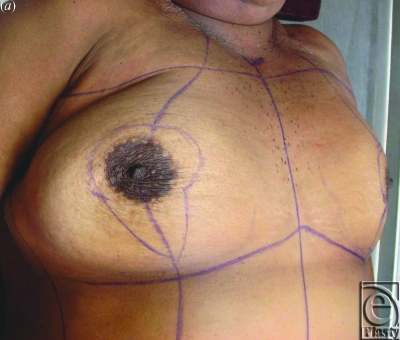

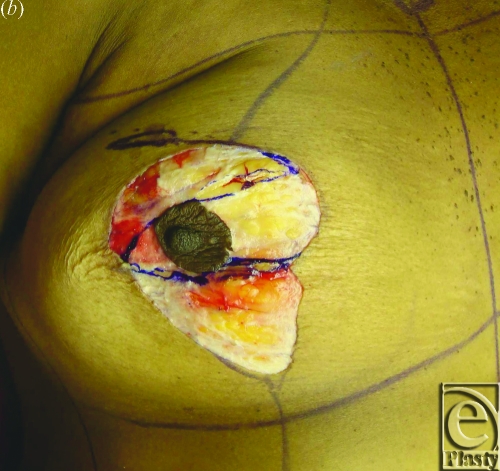

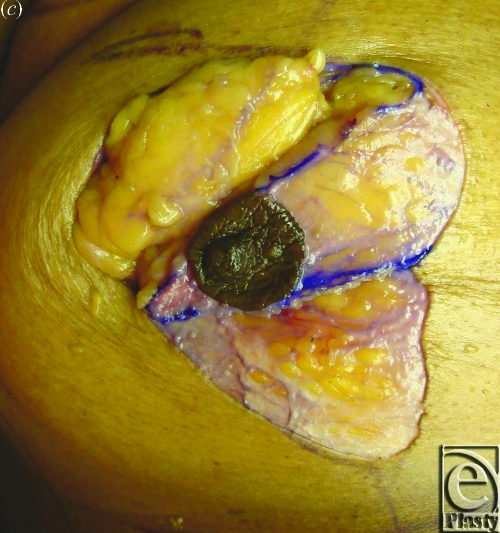

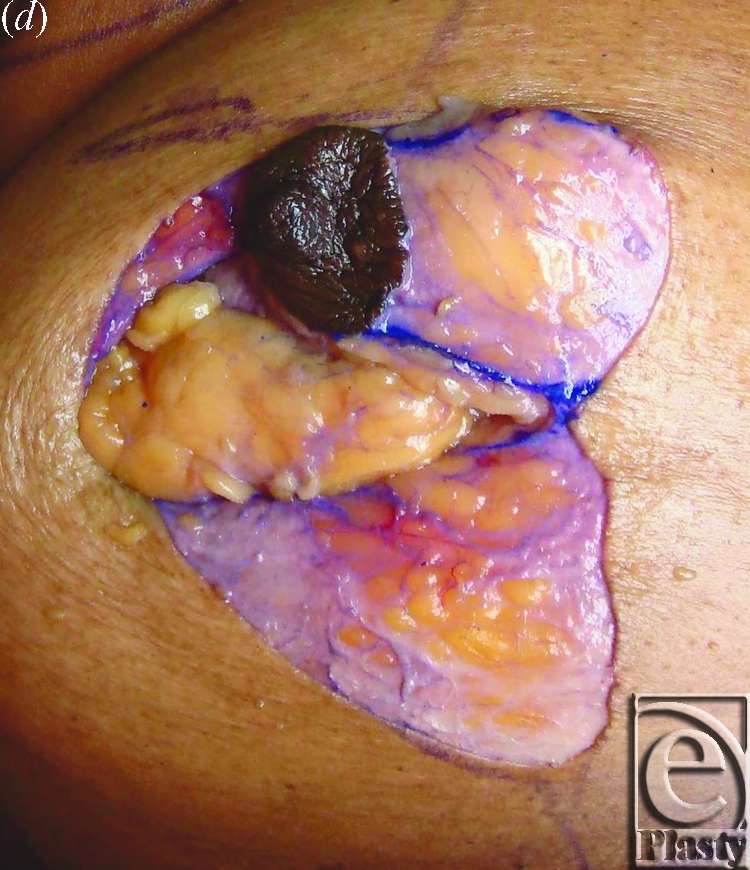

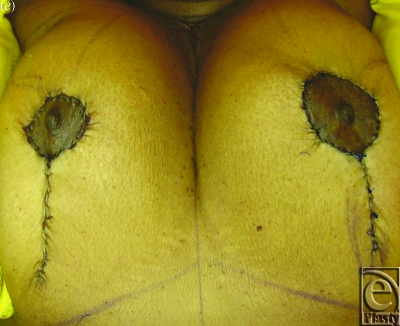
Vertical approach to Z-mammaplasty in fresh cadaver demonstrating: (*a*) skin markings, (*b*) incision, (*c*) intraoperative marking of planned glandular Z-plasty, (*d*) flap transposition carrying the nipple superiorly, and (*e*) closure of incisions.

**Figure 2:**
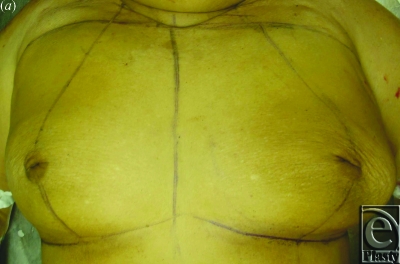

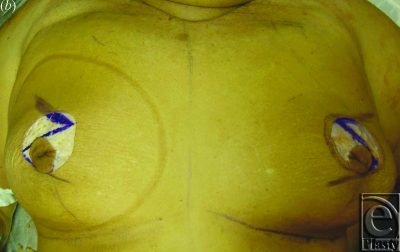

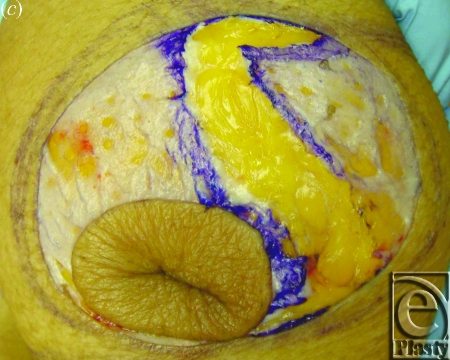

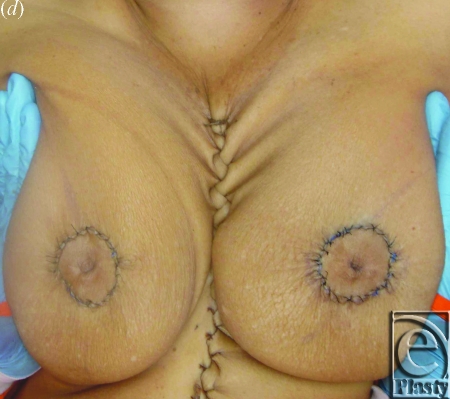
Periareolar approach to Z-mammaplasty demonstrating: (*a*) skin markings, (*b*) incision, (*c*) intraoperative marking of planned glandular Z-plasty, and (*d*) purse-string closure after flap transposition carried the nipple superiorly.

The breasts were approached either using a short vertical skin incision as described by Lejour and Abboud^6^ or via a periareolar skin incision. Surgical steps included skin incision, deepithelialization, and intraoperative marking of the planned glandular Z-plasty. The angles were chosen depending on breast size, shape, and degree of ptosis. The gland was then incised according to the form of the Z; flaps were undermined and transposed; and the inferior flap carried the nipple superiorly. For left-sided breasts, the Z was a mirror image of the right. For the short vertical skin incision group, the flaps were fully transposed. For the periareolar skin incision group, the flaps were partially transposed such that the superior flap was inset under the inferior Nipple bearing flap; this provided auto-augmentation. After glandular transposition of varying degrees and inset, the skin incision was then closed in the usual fashion. Photographs were taken to compare the breast appearance before and after the procedure (Figs 1 and 2), and the overall aesthetic appearance was graded on a scale from 1 (*worst*) to 5 (*best*).

## RESULTS

The average age was 71 ± 6.9 years and the average duration of the procedure was 58 ± 6.1 minutes per breast, including markings and measurements. Nine vertical short scar incisions were used, and 6 periareolar incisions were used. The average breast width preoperatively was 28.5 ± 4.7 cm while postoperatively was 26.7 ± 3.2 cm (*P* = .009). The average breast length preoperatively was 25 ± 6.6 cm while postoperatively was 21.8 ± 4.3 cm (*P* = .005). The average breast height preoperatively was 10.7 ± 3.7 cm while postoperatively was 9.5 ± 2.9 cm (*P* = .02). The average degree of ptosis preoperatively was 1.9 ± 0.9, mild to moderate ptosis, while postoperatively was 0.3 ± 0.5 (*P* < .0001), no ptosis. The average areolar diameter preoperatively was 5.7 ± 1.8 cm while the postoperatively was 4.5 ± 0.5 cm (*P* = .025). The average sternal-nipple distance preoperatively was 25.2 ± 3.2 cm while postoperatively was 19.9 ± 1.8 cm (*P* < .0001). The average fold-nipple distance preoperatively was 10.5 ± 3.8 cm while postoperatively was 9.5 ± 2.9 cm (*P* = .028). The average midline-nipple distance preoperatively was 15.1 ± 2.5 cm while postoperatively was 12.7 ± 1.8 cm (*P* = .0002). The average sternal notch-point A distance preoperatively was 18.8 ± 1.3 cm while postoperatively was 18.7 ± 1.5 cm (*P* = .33). The average vertical incision length preoperatively was 9.0 ± 2.3 cm while postoperatively was 7.6 ± 2.2 cm (*P* = .0004). The average distance above the fold where the vertical incision terminated preoperatively was 3.5 ± 2.4 cm while postoperatively was 3.4 ± 2.9 cm (*P* = .89). The average length of periareolar incision preoperatively was 20.6 ± 1.4 while postoperatively was 13.0 ± 1.5 (*P* < .0001). Table 1 summarizes the aforementioned findings.

The inferior limb transposition moved the nipple superiorly and medially closer to the sternal notch and sternum midline an average of 5.3 ± 2.2 cm and 2.4 ± 1.7 cm, respectively. The average Z-plasty degree was 34.5 ± 8.2°. The average central limb length 8.7 ± 2.1 and average pedicle width was 5.4 ± 0.8 cm. An average of 1.8 perforators greater than 1 mm in diameter were visualized and divided to freely mobilize the inferior limb of the Z (ie, superior-medial pedicle with nipple). Buttress support of the nipple was accomplished by the caudal transposition of the superior Z-plasty flap and its inset below the nipple. A Benelli block technique was used to create a purse-string suture in the periareolar group before final skin closure. Finally, the average overall aesthetic result was 3.3 ± 0.9; the average vertical scar aesthetic result was 3.0 ± 0.8; and the average periareolar aesthetic result was 3.6 ± 1.0 (*P* = .2). Table 2 summarizes the aforementioned findings.

## DISCUSSION

The Z-plasty is a fixture in the plastic surgeon's armamentarium for manipulation and control of skin and tissue. It has been utilized throughout history to counteract surgical problems ranging from burn contracture to scar revision.^7^ The basis is a fixed geometric pattern, which can reliably reorient tissue based on stable vascular supply. Use of the Z-plasty has been described in reorientation of mammaplasty skin scars and contractures but never previously utilized for glandular modification.^8^,^9^ In addition, lack of consensus regarding a single superior mammaplasty technique has led to multiple skin and glandular modifications.^10^ In search of a new approach to lifting the breast, we considered utilizing the classic Z-plasty. We aim to apply this reconstructive technique to the manipulation and reorientation of breast tissue to achieve both lift and support for the ptotic breast.

For patient safety and ethical reasons, novel cosmetic surgery techniques ideally should be tested in the experimental model prior to in vivo attempts. Herein was our motivation to test the concept of a glandular Z-plasty against fresh human cadaver breasts. Our goal was to introduce the concept, its principles, and to determine whether it is anatomically and surgically feasible and reasonable. Our results demonstrate that glandular Z-mammaplasty is indeed feasible within a reasonable time frame of less than 1 hour per breast. This time frame will likely be dependent on surgeon experience with this technique in addition to longer time requirements in the living patient for matters of hemostasis. With regard to our surgical aim to create lift, the grade of ptsosis was statistically significantly improved, with the nipple moving superiorly an average of 5.3 cm in our study group; the overall aesthetic result was judged to be 3.3 of 5-point scale. In addition, the z-mammaplasty technique was accessible and possible with both skin patterns.

Theoretically, a Z-plasty will reorient and lengthen the central limb of the breast, which would be counter-productive to the goal of a lift. We did not record any lengthening effect; in fact, the breast was determined to be smaller in all dimensions, including width, length, and height. By planning the inferior flap of the Z-plasty to include the nipple, a superomedial pedicle is naturally created, and upon transposition is superiorly rotated into an elevated position. In addition, the superior Z-plasty flap is inset in such a way that it acts as internal support or buttress.

The glandular Z-plasty, a basic and intuitive flap design for plastic surgeons, is feasible and reasonable in human female cadaveric breasts. This novel technique is primarily designed to address ptosis of the nipple-areolar complex (NAC) position (ie, mastopexy). Reduction mammaplasty can also be achieved by simply designing an elliptical glandular excision in place of the central limb of the Z-plasty. The glandular Z-flaps still transpose to close the defect after central limb elliptical excision of tissue.

The skin pattern design, weather wise, vertical, or periareolar, is essentially independent of the Z-plasty glandular rearrangement. What makes this technique different is that the Z-plasty is not in the skin, but within the gland itself. The inferior limb of the Z-plasty elevates the NAC after transposition of the glandular flaps and then the skin can be addressed by any chosen design pattern, wise, vertical, or periareolar.

For these reasons, we offer this concept as a novel approach in mastopexy and recommend further investigation.

## CONCLUSION

Clearly, there is a difference between a concept and proven technique, and much remains to be elucidated about the glandular Z-mammaplasty concept. Several questions remain before this surgical technique can be translated into the clinical and surgical realm. For example, can reduction mammaplasty be planned similarly or jointly, perhaps by excising the tissue from the superior wing of the Z-mammaplasty to reduce overall breast tissue? Will the technique work in conjunction with prosthetic breast augmentation? Is the aesthetic result acceptable in live patients with functional success?

It has become evident that the Z-mammaplasty is a surgically feasible procedure with the results of lifting nipple and breast tissue and creating an internal buttress using the transposed superior breast flap. It remains to be seen whether this technique will serve applicable to the current plastic surgeon's approach to aesthetic breast surgery. Further investigation is warranted.

## Figures and Tables

**Table 1:** Pre- and postoperative measurement comparisons

	Total			Vertical Incision			Periareolar Incision		
Average	Preoperative	Postoperative	*P*	Preoperative	Postoperative	*P*	Preoperative	Postoperative	*P*
Age, y	71 ± 6.9		N/A	71.7 ± 8.2		N/A	69.3 ± 5.2		N/A
Width, cm	28.5 ± 4.7	26.7 ± 3.2	.0089	31.1 ± 3.7	28.6 ± 2.2	.019	25.3 ± 3.8	24.5 ± 2.7	.26
Length, cm	25 ± 6.6	21.8 ± 4.3	.005	27.5 ± 6.8	24.1 ± 4.7	.01	22.2 ± 5.5	19 ± 1.3	.15
Height, cm	10.7 ± 3.7	9.5 ± 2.9	.02	12.8 ± 3.6	11.0 ± 2.9	.01	8.2 ± 2.1	7.7 ± 1.9	.5
Degree ptosis	1.9 ± 0.9	0.3 ± 0.5	<.0001	1.9 ± 0.9	0 ± 0	.0015	2 ± 0.9	0.7 ± 0.5	.0014
Areolar diameter, cm	5.7 ± 1.8	4.5 ± 0.5	.025	6.8 ± 1.8	4.7 ± 0.3	.014	4.3 ± 0.5	4.3 ± 0.5	1
Sternal-nipple, cm	25.2 ± 3.2	19.9 ± 1.8	<.0001	26.7 ± 3.5	21 ± 1.8	.0005	23.5 ± 2.0	18.7 ± 0.5	.0037
Fold-nipple, cm	10.5 ± 3.8	9.5 ± 2.9	.028	12.8 ± 3.6	11 ± 2.9	.006	7.8 ± 1.8	7.7 ± 1.9	.79
Midline-nipple, cm	15.1 ± 2.5	12.7 ± 1.8	.0002	16.1 ± 3.0	13.4 ± 2	.001	14 ± 1.1	11.8 ± 1.2	.05
Point A distance	18.8 ± 1.3	18.7 ± 1.5	.33	18.9 ± 1.6	18.7 ± 1.9	.35	18.7 ± 1	18.7 ± 1	1
Length of incision	14.4 ± 6.3	10.1 ± 3.4	.0005	9.0 ± 2.3	7.6 ± 2.2	.0004	20.7 ± 1.4	13 ± 1.5	<.0001
Distance above fold	1.9 ± 2.5	1.8 ± 2.7	.89	3.5 ± 2.4	3.4 ± 2.9	.89	N/A	N/A	N/A

**Table 2:** Comparison of vertical and periareolar final results

Average	Total	Vertical Incision	Periareolar Incision
Z-plasty degree	34.5 ± 8.2	37.6 ± 6.4	30.8 ± 9.2
Central limb length, cm	8.7 ± 2.1	9.9 ± 2	7.3 ± 1.4
Pedicle width, cm	5.4 ± 0.8	5.9 ± 0.4	4.9 ± 0.7
Pedicle undermining, cm	8.1 ± 2.9	9.3 ± 3.5	6.7 ± 0.5
Aesthetic result	3.3 ± 0.9	3.0 ± 0.8	3.7 ± 1
	Distance traveled by nipple, cm		
Sternal-nipple	5.3 ± 2.2	5.7 ± 2.2	4.8 ± 2.3
Midline-nipple	2.4 ± 1.7	2.6 ± 1.3	2.2 ± 2.1
